# Application of xCELLigence real-time cell analysis to the microplate assay for pertussis toxin induced clustering in CHO cells

**DOI:** 10.1371/journal.pone.0248491

**Published:** 2021-03-15

**Authors:** Lidice Bernardo, Lucas Corallo, Judy Caterini, Jin Su, Lucy Gisonni-Lex, Beata Gajewska

**Affiliations:** Department of Analytical Sciences, Sanofi Pasteur, Toronto, ON, Canada; Xiangtan University, CHINA

## Abstract

The microplate assay with Chinese Hamster Ovary (CHO) cells is currently used as a safety test to monitor the residual pertussis toxin (PT) amount in acellular pertussis antigens prior to vaccine formulation. The assay is based on the findings that the exposure of CHO cells to PT results in a concentration-dependent clustering response which can be used to estimate the amount of PT in a sample preparation. A major challenge with the current CHO cell assay methodology is that scoring of PT-induced clustering is dependent on subjective operator visual assessment using light microscopy. In this work, we have explored the feasibility of replacing the microscopy readout for the CHO cell assay with the xCELLigence Real-Time Cell Analysis system (ACEA BioSciences, a part of Agilent). The xCELLigence equipment is designed to monitor cell adhesion and growth. The electrical impedance generated from cell attachment and proliferation is quantified via gold electrodes at the bottom of the cell culture plate wells, which is then translated into a unitless readout called cell index. Results showed significant decrease in the cell index readouts of CHO cells exposed to PT compared to the cell index of unexposed CHO cells. Similar endpoint concentrations were obtained when the PT reference standard was titrated with either xCELLigence or microscopy. Testing genetically detoxified pertussis samples unspiked or spiked with PT further supported the sensitivity and reproducibility of the xCELLigence assay in comparison with the conventional microscopy assay. In conclusion, the xCELLigence RTCA system offers an alternative automated and higher throughput method for evaluating PT-induced clustering in CHO cells.

## Introduction

*Bordetella pertussis* bacterium is the cause of whooping cough. The main virulence factor of *B*. *pertussis* is pertussis toxin (PT), although dermonecrotic toxin, tracheal cytotoxin, adenylate cyclase toxin, and cell binding proteins such as filamentous hemagglutinin, fimbriae, and pertactin are also involved in pathogenesis [1]. Whole cell and acellular vaccines are available to prevent the disease. The whole cell vaccine consists of whole cell pertussis inactivated by heat or formalin. The acellular vaccines are composed of purified pertussis antigens (such as pertussis toxoid, filamentous hemagglutinin, fimbriae and/or pertactin) at different concentrations and combinations. Pertussis toxoid is the main component in all acellular vaccines [2].

Pertussis toxin belongs to the family of toxins with ADP-ribosylating activity that catalyzes the transfer of an ADP-ribose group from a NAD^+^ substrate to a cysteine residue located in the C-terminal region of the α-subunit of GTP binding proteins (G proteins) [3,4]. ADP-ribosylation then leads to uncoupling of G proteins from their receptors, which is believed to be responsible for several biological effects associated with PT [5,6]. For example, ADP-ribosylation of G inhibitory protein inactivates the α-subunit and thus it is unable to inhibit adenylyl cyclase activity. Due to its potent toxic effect, PT needs to be inactivated to pertussis toxoid by chemical treatment to ensure vaccine safety. The microplate assay using Chinese Hamster Ovary (CHO) cells with microscopy scoring based on cell clustering is currently used to determine the residual amount of PT activity in acellular pertussis components prior to vaccine formulation [7–9]. The assay is based on the findings of Hewlett et al. (1983) that the exposure of CHO cells to pertussis toxin results in a concentration-dependent clustering response [10]. The mechanism responsible for the clustering effect on CHO cells is not fully understood. Toxin internalization is mediated by the B domain which binds to various receptors on the surface of eukaryotic cells and allows the internalization of the active S1 subunit located in the A domain [11,12]. After internalization, the toxin undergoes retrograde transport to the endoplasmic reticulum through the Golgi apparatus [13–15]. Burns and his collaborators (1987) have shown that the clustering effect is related to the ADP-ribosylation of a 41-kDa protein, which supports the suitability of the CHO clustering assay to detect the enzymatic activity of PT, but the events after the ADP-ribosylation that lead to the clustering cell morphology remain to be elucidated [16,17]. The microplate assay is designed as a simple titration assay in which CHO cells are exposed to acellular pertussis samples or reference PT in a range of dilutions [7]. After incubation, the wells are observed using an inverted light microscope and the CHO cells are scored for clustering. The highest dilution of the test sample that results in clustering represents the endpoint titer. Analysis of test sample against the reference PT preparation provides a semi-quantitative estimate of PT content.

Although the CHO cell assay used to detect PT is very sensitive [18], the microscopy reading is laborious and prone to subjective scoring by the analyst. Reading of the plates by two analysts is often carried out to reduce the subjectivity of the visual scoring [19,20]. In the present work, we have explored the feasibility of replacing the microscopy scoring step of the microplate CHO cell assay with the xCELLigence Real-Time Cell Analysis system for the readout (ACEA BioSciences, part of Agilent) [21–29]. The xCELLigence is designed for monitoring cell adhesion and growth. The system uses microplates with gold electrodes on the bottom of the wells, such that an electric potential can be applied across a conductive fluid within wells. Naturally, as more cells adhere to a well, the degree to which electrons can flow freely across the established potential is reduced. The xCELLigence analyzer refers to this phenomenon as *impedance* and translates the measured impedance into a unitless readout called cell index [24]. We hypothesized that the disruption in the integrity of the CHO cell monolayer caused by PT-induced clustering can be detected as a decrease in the cell index readout compared to the intact CHO monolayer of untreated CHO cells.

Based on the finding presented herein, the xCELLigence system is suitable to detect the morphological changes induced by PT in CHO cells and the results aligned with the current microscopy scoring. The xCELLigence technology could be adopted as a replacement for the microscopy readout as a more objective and higher throughput system for the microplate CHO cell assay.

## Material and methods

### Reagents and cells

CHO-K1 cells (CCL-61; American Type Culture Collection) were stored in liquid nitrogen at cell passage number nine. Cells were thawed and cultured into 75 cm^2^ tissue culture flasks with complete growth medium [Kaighn’s modified Ham’s F-12 medium (F12K; Gibco), 10% fetal bovine serum (Hyclone), 200 mM L-glutamine (Gibco) and 1X antibiotic/antimycotic (Gibco)] four days prior to the experiment. On the day of experiment, 80–90% confluent cell monolayers were trypsinized [0.25% trypsin-EDTA (Gibco)] and diluted in complete growth medium to 2.5 x 10^4^ or 5 x 10^4^ cell/mL for CHO cell assay by microscopy or xCELLigence, respectively. Culture conditions were 36°C to 37°C with an atmosphere of 5% CO_2_.

Biological reference preparation of pertussis toxin (BRP PT, referred to here as standard PT) calibrated in international units [Batch 1; European Directorate for the Quality of Medicines and Healthcare (EDQM), 0.050 mg equivalent to 7500 IU/vial] [30] was reconstituted in 1 mL of water for injection (Gibco) as per manufacturer recommendation. An in-house preparation of PT (0.369 mg/mL) was obtained from the Quality Control Department at Sanofi Pasteur Canada and stored at -20°C.

Anti-PT monoclonal antibody clone LP12.2 (Sanofi Pasteur) was used for the neutralization assay. Irrelevant, non-PT specific antibody clone 1–24.1.20 (Sanofi Pasteur) was used as a control.

Genetically detoxified PT (gdPT) (Sanofi Pasteur, Canada) was used as the sample for assay development [31,32]. The gdPT samples were also evaluated with spiking of the native PT. The spiked gdPT samples were prepared by adding 10 μL of the standard PT stock diluted to the desired spiking concentration in complete growth medium to 490 μL of sample at 50 μg/mL. Unspiked samples were prepared by adding 10 μL of phosphate buffered saline to 490 μL of sample at 50 μg/mL as control.

### Microplate CHO cell assay (microscopy readout)

The microplate CHO cell clustering assay was performed as previously described [7] with some modifications. In brief, samples (pre-diluted to 50 μg/mL of protein), standard PT (reference standard in IU/mL) and in-house preparation of PT toxin were diluted two-fold in 96 well plates (25 μL) and incubated with 200 μL of CHO cells (5 x 10^3^ cells/well) for 48 hours at 37°C and 5% CO_2_. The plate was observed under an inverted light microscope to score each well as positive or negative for cell clustering. As per in-house method, a well was considered positive when ≥ 80% of the cells in the well exhibited clustering by visual observation and negative when cell clustering was < 80%. The concentration at the highest sample or reference standard dilution exhibiting ≥ 80% cell clustering was considered as the endpoint concentration. To calculate the PT concentration in the sample (IU/μg of protein), the PT reference standard endpoint concentration was divided by the sample endpoint concentration.

### Microplate CHO cell assay with xCELLigence readout

Samples, standard PT and in-house PT were prepared in the xCELLigence E-Plate (ACEA BioSciences, VIEW 96 PET plate) as described above for the microplate CHO cell assay with microscopy readout. Additional 25 μL of complete growth medium was added to all wells to reach the 50 μL recommended for blanking the plates. The E-plate was engaged into the xCELLigence (ACEA BioSciences, SP or MP model) and background measurement of the wells were recorded before adding 100 μL of CHO cells (5 x 10^3^ cells/well). Cells were allowed to settle for 30 minutes at room temperature before the E-plates were re-engaged onto the xCELLigence analyzer and incubated for 48 h at 36°C to 37°C and 5% CO_2_. Impedance values were recorded every hour and the time point closer to 48 h (above or below) was used for data analysis.

### PT neutralization assay

A fixed concentration of PT known to induce cell clustering [1 IU/mL (6.7 ng) and 6.8 ng/mL for standard PT and in-house PT, respectively] (25 μL/well in the E-plate) was pre-incubated with equal volume of anti-PT antibody LP12.2 serially diluted five-fold (285.2, 57.0 and 11.4 μg/mL; molar ratios of PT to antibody of 1 to 30000, 1 to 6000 and 1 to 1000, respectively) for 30 min at 37°C and 5% CO_2_. Incubation of pertussis toxin with irrelevant (non PT-specific) antibody clone 1–24.1.20 (285.2 μg/mL) and complete growth medium were used as negative neutralization controls. After incubation of PT and antibody mixtures, a background measurement of the E-plate was taken by the xCELLigence analyzer before adding the CHO cells (5 x 10^3^ cells/well). Incubation and data collection were performed as described above in “Microplate CHO cell assay with xCELLigence readout”.

### Statistical analysis

Comparison of cell index values from cells treated with variable concentrations of PT versus cell control was performed by the Kruskal-Wallis test followed by Dunn’s multiple comparison test (GraphPad Prism v. 8.0).

## Results

### Suitability of the xCELLigence system to detect presence of pertussis toxin in CHO cells

**Fig 1:**
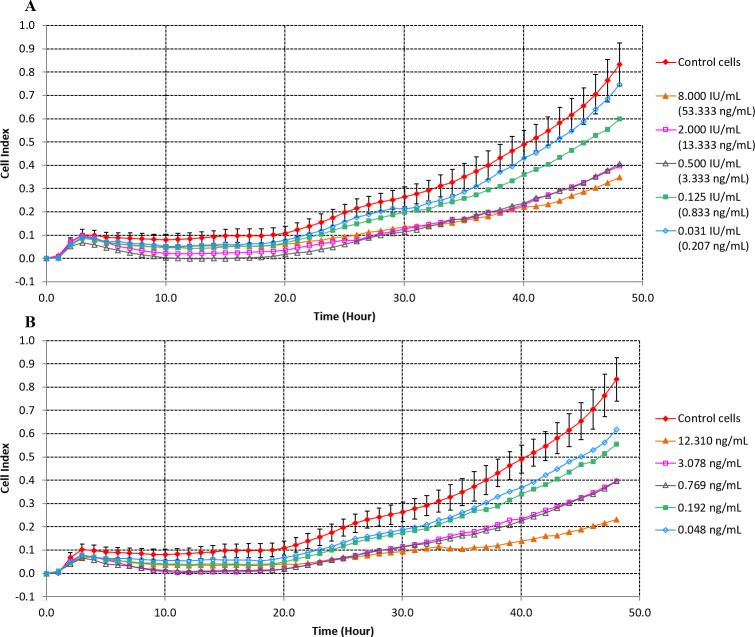
Cell index in real time after treatment of CHO cells with variable concentrations of pertussis toxin. (A) Treatment with standard pertussis toxin. (B) Treatment with in-house pertussis toxin. Control cells were incubated with culture medium only. Results of one representative experiment are shown [n = 1 for all pertussis treatment and n = 6 for the cell controls (Average of cell control represented)]. Error bars represent standard deviations.

In order to evaluate whether PT-induced clustering in CHO cells would be reflected as a decrease in cell index readouts by xCELLigence, varying concentrations of PT were incubated with CHO cells (negative control wells received only complete growth medium instead of PT). Cell index readouts were recorded over a 48 h incubation period. As shown in Fig 1A (standard PT) and 1B (in-house PT), an inverse dose response correlation between PT concentration and cell index was observed. The curve for the lowest PT concentration tested was very close to the cell control curve, while the curve corresponding to the highest PT concentration showed the lowest cell index value. This dose response correlation was more evident at 48 h than at earlier time points, where the curves frequently overlapped. These results confirm our hypothesis that the disruption in the integrity of the CHO cell monolayer caused by PT-induced clustering is detected as a decrease in the cell index readout compared to the control cells.

### Specificity of the cell index readout to the pertussis toxin activity in CHO cells

**Fig 2:**
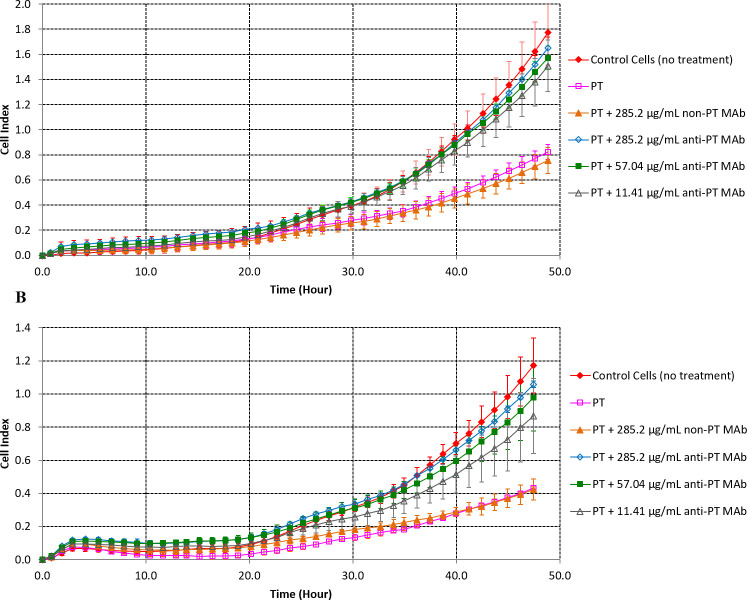
Cell index in real time after treatment of CHO cells with pertussis toxin alone or preincubated with anti-pertussis toxin or irrelevant non-PT antibodies. (A) Standard pertussis toxin (1 IU/mL = 6.7 ng/mL), average of 2 or more replicates are shown for each condition. (B) In-house pertussis toxin (6.8 ng/mL), average of 3 or more replicates are shown for each condition. Antibodies were serially diluted to PT to antibody molar ratios of 1 to 30000 (285.2 μg/mL), 1 to 6000 (57.0 μg/mL) and 1 to 1000 (11.4 μg/mL). Error bars represent standard deviations.

To confirm that the decrease in cell index readouts is specifically related to the PT activity on CHO cells, a neutralization assay was conducted with anti-PT specific neutralizing antibody LP12.2 that binds to the S3/S4 dimer of the B-oligomer, responsible for the binding to receptors on target cells [31,32]. As expected, the cell index was highest for the cell control at 48 h, while PT treatment of CHO cells without anti-PT antibodies or with an irrelevant antibody notably decreased the cell index readout (Fig 2). In contrast, preincubation of PT with anti-PT neutralizing antibodies greatly increase the cell index, with values falling just below those of the control cells at 48 h. Similar results were obtained for both standard PT (Fig 2A) and in-house PT (Fig 2B). These results demonstrate that the decrease in cell index readout is specific to the presence of PT in the wells.

### Positive/Negative threshold based on cell index

**Fig 3:**
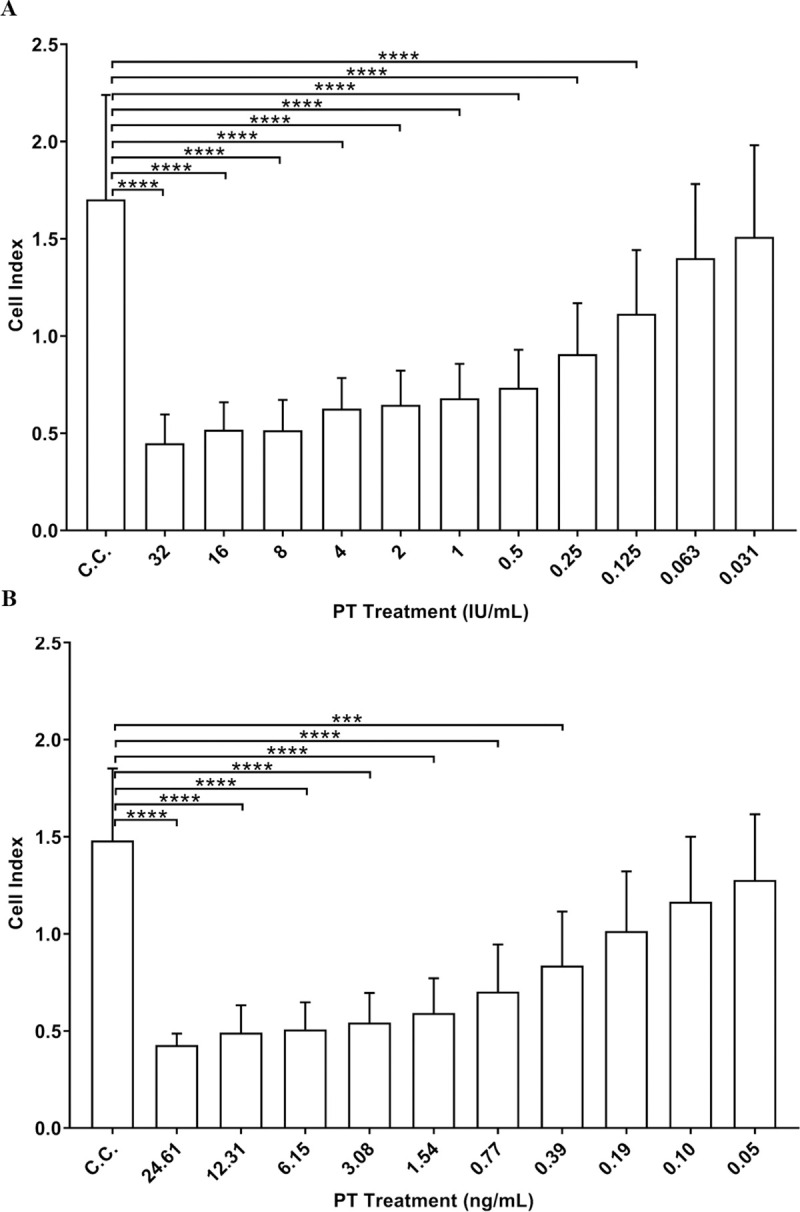
Mean cell index of pertussis toxin treatments versus cell control. (A) Standard pertussis toxin results with at least n = 15 for each toxin treatment and n = 302 for cell control (C.C.). (B) In-house pertussis toxin with at least n = 7 for each toxin treatment and n = 60 for C.C. condition. Statistical analysis was performed by Kruskal-Wallis test followed by Dunn’s multiple comparison test (GraphPad Prism v. 8.0) (**** p <0.0001, *** p < 0.001). Error bars represent standard deviations.

To define the assay threshold for a positive response by xCELLigence cell index readout, data collected from multiple experiments (n = 33 for standard PT and n = 9 for in-house PT) were pooled and compared to determine the cell index values among the PT treatments that were significantly different from the cell control cell index (Fig 3). The mean cell index response at the lowest PT concentration significantly reducing the cell index compared to the cell control was used to set the positive threshold for the assay. Instead of using the cell index values to set the threshold, the percentage response of reference standard cell index to mean cell control cell index (Relative cell index) was calculated within each plate to account for the assay to assay variability on the cell index readout.

As shown in Fig 3 and Tables 1 and 2, the lowest PT treatment that significantly decreased the cell index compared to the cell control had mean Relative cell index of 61% and 57% for standard PT and in-house PT, respectively. The highest PT treatment showing non-significant decrease in the cell index readout compared to the cell control had mean Relative cell index of 73% and 70% for standard PT and in-house PT, respectively. The assay threshold for a positive response was then defined as Relative cell index ≤ 65%, based on the Relative cell index associated with PT concentrations inducing significant and non-significant decrease of the cell index compared to the mean cell index of the cell control wells.

**Table 1:** Relative cell index at each standard PT concentration.

PT treatment (IU/mL) [ng/mL]	32 [213.3]	16 [106.7]	8 [53.3]	4 [26.7]	2 [13.3]	1 [6.7]	0.5 [3.3]	0.25 [1.7]	0.125 [0.8]	0.063 [0.4]	0.031 [0.2]
**Statistical Comparison Cell Index PT Treatment vs Cell Index Cell Control**^**a**^	****	****	****	****	****	****	****	****	****	ns	ns
**Mean Relative Cell Index (%)**^**b**^	35	37	32	34	35	37	43	50	61	73	86

^a^Cell index collected from multiple experiments (n = 33) were compared by Kruskal-Wallis test followed by Dunn’s multiple comparison test, p < 0.05 was considered significant.

****: p ≤ 0.0001; ns: Non-significant.

^b^PT treatment Cell Index/Cell Control Cell Index x 100.

**Table 2:** Relative cell index at each concentration of in-house PT.

PT treatment (ng/mL)	49.22	24.61	12.31	6.15	3.08	1.54	0.77	0.39	0.19	0.10	0.05
**Statistical Comparison Cell Index Treatment vs Cell Index Cell Control**^**a**^	****	****	****	****	****	****	****	**	ns	ns	ns
**Mean Relative Cell Index (%)**^**b**^	28	33	34	35	38	41	48	57	70	80	83

^a^Cell index collected from multiple experiments (n = 9) were compared Kruskal-Wallis test followed by Dunn’s multiple comparison test, p < 0.05 was considered significant.

****: p ≤ 0.0001

**: p ≤ 0.01; ns: Non-significant.

^b^PT treatment cell index/Cell Control cell index x 100.

### Comparing xCelligence versus microscopy readouts

**Fig 4:**
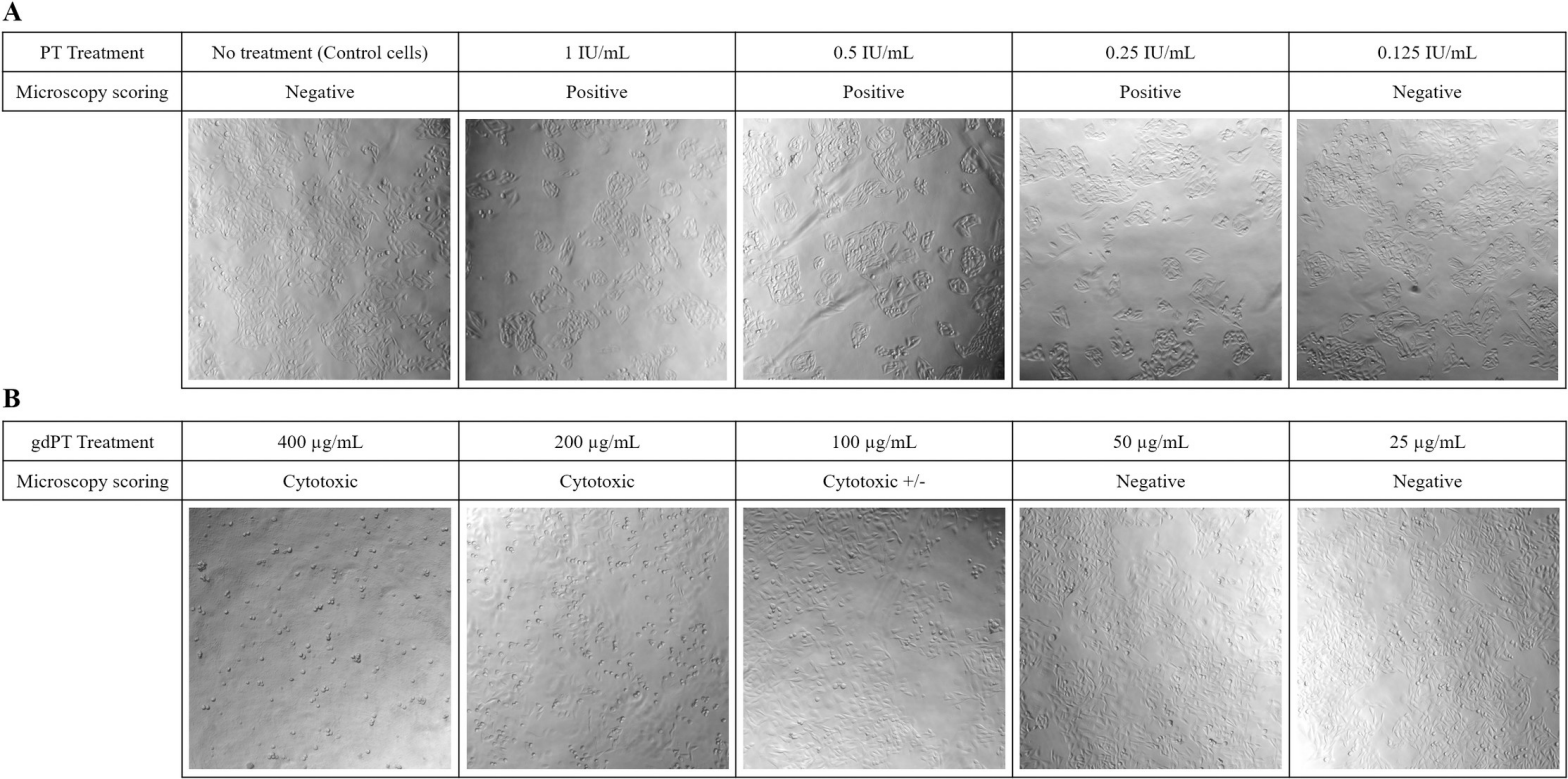
Modifications to the cell layer by microscopy method 48 h after treatment with standard PT or gdPT. (A) Control cells versus cells treated with 1 IU/mL to 0.125 IU/mL of PT (corresponding to Table 3, experiment 3, replicate 2) and the scoring of each well by microscopy (positive: ≥ 80% cell clustering per well; negative: < 80% cell clustering). (B) CHO cells exposed to concentrations of gdPT from 400 μg/mL to 50 μg/mL. Cytotoxicity +/- indicates low visible level of cell death.

To compare microscopy and xCELLigence readouts, experiments were performed where duplicate 96-well culture plates with a standard PT dilution series were set up alongside an E-Plate. The CHO cell clustering assay (microscopy readout) and the xCELLigence were performed as described earlier, with the same preparation of PT dilutions and CHO cells. After 48 h of incubation (regular reading time for the microscopy method), the 96-well culture plates were examined under the microscope to score each well as positive for clustering (≥ 80% cell clustering per well) or negative for clustering (< 80% cell clustering per well). The xCELLigence analysis was performed at the time point closest to 48h by calculating the Relative cell index for each well (Relative cell index = treatment cell index ÷ mean cell control cell index x 100). Wells with Relative cell index ≤ 65% were considered as positive for PT activity and wells with Relative cell index > 65% were considered as negative. As presented in Table 3, results showed a median endpoint concentration of 0.250 IU/mL when the plates were visually examined under the microscope, and a median endpoint concentration of 0.125 IU/mL by xCELLigence analysis. Fig 4A shows representative pictures of the appearance of the cells in the standard 96-well culture plates after treatment with 1 IU/mL to 0.125 IU/mL of standard PT, taken by microscopy at the time of reading (48 h). The xCELLigence appeared to be slightly more sensitive than the microscopy readout, however, the values obtained by both methods are within a two-fold dilution, which is within the reported variability for the assay [7]. The variability of the microscopy assay was reported by Gillenius and his collaborators in 1985, who repeatedly titrated samples by two-fold dilution steps and showed inter-assay endpoint variations of plus or minus one dilution step from the median titers [7]. All endpoint concentrations by xCELLigence were also within one two-fold dilution of the median value, indicating the variability of the xCELLigence technique is similar to the variability for microscopy method [7].

**Table 3:** Scoring cells treated with standard pertussis toxin concentrations for CHO cell clustering by microscopy and xCELLigence.

Method	Experiment	Standard PT (IU/mL) ^a^								PT Endpoint Conc (IU/mL)	PT Median endpoint Conc (IU/mL) (expected variability range)
		2	1	0.5	0.25	0.125	0.063	0.031	0.016		
**Microscopy**	1	+	+	+	+	-	-	-	-	0.25	0.25 (0.125–0.5)
	2	+	+	+	+	-	-	-	-	0.25	
	3-replicate 1	+	+	+	+	+	-	-	-	0.125	
	3-replicate 2	+	+	+	+	-	-	-	-	0.25	
	4-replicate 1	+	+	+	+	+	-	-	-	0.125	
	4-replicate 2	+	+	+	+	+	-	-	-	0.125	
	5-replicate 1	+	+	+	+	-	-	-	-	0.25	
	5-replicate 2	+	+	+	+	-	-	-	-	0.25	
**xCELLigence**	1	+	+	+	+	+	-	-	-	0.125	0.125 (0.063–0.25)
	2	+	+	+	+	+	-	-	-	0.125	
	3-replicate 1	+	+	+	+	+	-	-	-	0.125	
	3-replicate 2	+	+	+	+	+	-	-	-	0.125	
	4-replicate 1	+	+	+	+	+	-	-	-	0.125	
	4-replicate 2	+	+	+	+	+	+	-	-	0.063	
	5-replicate 1	+	+	+	+	+	-	-	-	0.125	
	5-replicate 2	+	+	+	+	+	-	-	-	0.125	

^a^ Results after 48 h of incubation.

“-”: Negative for cell clustering (Microscopy: ≥ 80% cell clustering per well; xCELLigence: Relative cell index ≤ 65%).

“+”: Positive for cell clustering (Microscopy: < 80% cell clustering per well; xCELLigence: Relative cell index > 65%).

Endpoint Conc: Endpoint concentration, last concentration of standard PT to show a positive response.

Expected variability range: Plus or minus one two-fold dilution of the median.

### Titrating unspiked and PT spiked gdPT samples by xCELLigence and microscopy CHO cell assay methods

To assess the feasibility of the xCELLigence CHO cell assay for samples that contain none or low levels of PT activity, genetically detoxified PT (gdPT) samples, unspiked or spiked with standard PT were evaluated in parallel by xCELLigence and microscopy methods. The gdPT is produced from a genetically engineered *B*. *pertussis* strain that contains two genetic modifications within the S1 subunit of the toxin (Arg9Lys and Glu129Gly) that abolish the enzymatic activity of the protein [31,32]. The gdPT sample matrix is equivalent to the pertussis toxin matrix without the toxin activity, which provides the right environment for representative measurements of the PT concentrations in the spiked samples. Samples were pre-diluted to 50 μg/mL and tested unspiked and spiked with either 2.5 or 10 IU/mL (16.7 or 66.7 ng/mL, respectively) of standard PT. The standard PT spiking concentration of 10 IU/mL into 50 μg/mL sample of gdPT represents the maximum amount of pertussis considered safe for humans for a 10 μg human dose of gdPT (target value of 2 IU/human dose/10 μg of gdPT) [30,33,34]. The CHO assay set up for the xCELLigence and microscopy methods was performed as described above. After 48 h of incubation, cell index at the time point closest to 48 h were used to calculate the Relative cell index for each well of the E-Plate and determine the sample and standard PT endpoint concentrations (lowest concentration with Relative cell index ≤ 65%) by xCELLigence. Endpoint concentration for the microscopy method were determined the by visual scoring of the cell clustering under the microscope as described above. The PT concentration in each sample (IU/μg), was calculated by dividing the standard PT endpoint concentration by the sample endpoint concentration.

As shown in Table 4, the unspiked gdPT endpoint concentration was either below or at the first concentration of gdPT tested (< 50 μg/mL or 50 μg/mL). It should be noted that positive scoring at 50 μg/mL by xCELLigence are not indicative of CHO cell clustering but sample matrix cytotoxicity on the cells at high protein concentration, as observed by microscopy readout (Fig 4B) and also reported previously by others [7]. As shown in Fig 4B images, high degree of cell death is apparent at gdPT concentrations between 400 μg/mL and 100 μg/mL, while no cell clustering is observed at any gdPT concentration. Nonetheless, with the xCELLigence automatic readout considering cytotoxic effect as a positive readout, the maximum PT activity obtained was 0.0025 IU/μg of protein (equivalent to 0.025 IU for a gdPT dose of 10 μg) which is well below the levels of PT considered safe for humans [30,33,34]. In contrast, calculated PT concentrations in gdPT samples spiked with 2.5 or 10 IU/mL of standard PT by xcELLigence and microscopy methods were nearly 10 to 60 times higher than gdPT alone (refer to average results for gdPT unspiked and spiked in Table 4). PT concentration results for gdPT spiked samples support equivalent sensitivities for the xCELLigence and the microscopy methods, with identical average PT concentration detected in the 2.5 IU/mL PT spiked sample (0.03 IU/μg by both xCELLigence and microscopy) and very close average values for the 10 IU/mL PT spiked sample (0.134 IU/μg by xCELLigence and 0.120 IU/μg by microscopy).

**Table 4:** Pertussis toxin detection in gdPT samples unspiked and spiked with standard pertussis toxin by xCELLigence and microscopy readouts.

Sample	Experiment	Standard PT endpoint concentration (IU/mL)^a^		Sample endpoint concentration (μg/mL)^b^		Sample PT concentration (IU/μg)^c^		Average PT concentration (IU/μg) [IU/10 μg gdPT]^d^	
		xCELLigence	Microscopy	xCELLigence	Microscopy	xCELLigence	Microscopy	xCELLigence	Microscopy
gdPT Unspiked	1	0.125	NT	< 50.00	NT	< 0.0025	NT	<0.0023 [<0.023]	<0.0038 [<0.038]
	2	0.094	NT	50.00	NT	0.0019	NT		
	3	0.125	NT	50.00	NT	0.0025	NT		
	4	0.125	0.188	50.00	<50.00	0.0025	<0.0038		
	5	0.094	0.125	50.00	<50.00	0.0019	<0.0025		
	6	0.125	0.250	<50.00	<50.00	<0.0025	<0.0050		
gdPT Spiked (2.5 IU/mL = 16.7 ng/mL)	1	0.125	NT	6.25	NT	0.0200	NT	0.0300 [0.300]	0.0300 [0.300]
	2	0.094	NT	3.13	NT	0.0300	NT		
	3	0.125	NT	3.13	NT	0.0399	NT		
	4	0.125	0.188	3.13	6.25	0.0399	0.0300		
	5	0.094	0.125	3.13	6.25	0.0300	0.0200		
	6	0.125	0.250	6.25	6.24	0.0200	0.0400		
gdPT Spiked (10 IU/mL = 66.7 ng/mL)	1	0.125	NT	1.56	NT	0.0801	NT	0.1336 [1.336]	0.1201 [1.201]
	2	0.094	NT	0.78	NT	0.1204	NT		
	3	0.125	NT	0.78	NT	0.1603	NT		
	4	0.125	0.188	0.78	1.56	0.1603	0.1200		
	5	0.094	0.125	0.78	1.56	0.1204	0.0800		
	6	0.125	0.250	0.78	1.56	0.1603	0.1603		

^a^Mean of two replicates.

^b^gdPT concentration at the highest positive dilution (≤ 65% Relative cell index by xCELLigence and ≥ 80% clustering by microscopy) at 48 h, one replicate per experiment.

^c^Reference standard endpoint concentration/gdPT endpoint concentration.

^d^10μg of gdPT: Equivalent to one human dose. Levels of PT up to 2 IU/human dose are considered safe for humans [30,33,34].

In addition to the suitability of the assay to detect different levels of PT in comparison with the microscopy method, the xCELLigence results also indicate good inter-assay precision, with all endpoint concentrations for standard PT, unspiked gdPT and spiked gdPT samples within a two-fold dilution of each other across the experiments (Table 4). A variation within a two-fold dilution is acceptable for endpoint titration assays, as discussed above [7].

## Discussion

The CHO cell assay by microtitre plate is used to monitor residual PT content in *B*. *pertussis* vaccine components such as pertussis toxoid, filamentous hemagglutinin, fimbriae and pertactin. The CHO cell clustering test has been historically applied upstream in the manufacturing process to non-adjuvanted intermediates, due to the inherent cytotoxicity of the adjuvants on the CHO cells [35]. New CHO assays have recently been developed that overcome the adjuvant cytotoxicity either by dilution of the product or the use of cell culture transwell inserts [36]. However, as of 2020, the European Pharmacopoeia Commission has removed the requirement to test the adjuvanted drug products for residual PT [8,9]. The European Pharmacopoeia Monographs now consider the CHO cell assay testing of the pre-adsorbed drug substance (the active pharmaceutical ingredient before it is formulated into the drug product, where antigens are more concentrated than at the drug product stage) as the most effective approach for detection of pertussis toxin. It should be noted that as part of the same monograph revision, the requirement to perform Histamine Sensitization Test in mice and the test for irreversibility of pertussis toxoid were also deleted from the monographs on acellular pertussis vaccines. These updates were based on the results of two collaborative studies run under the auspices of the EDQM [20,36].

One of the main advantages of the CHO cell clustering assay is that it covers the internalization and translocation of the toxin from the cell membrane to the cytosol and its intracellular ADP-ribosylation activity, and therefore is considered comparable to the Histamine Sensitization Test [16,37,38]. The CHO cell assay is also very sensitive and detects lower levels of PT than Histamine Sensitization Test [17,18]. A major challenge with the current CHO cell assay methodology is that the scoring of PT induced clustering is dependent on subjective operator visual interpretation using light microscopy to determine the cut-off titre or concentration, and hence the final readout of the assay. High assay variability has also been reported, which has been attributed to the variety of reagents and methods used [30]. Of note, the reagents and CHO cells used for the CHO microplate assays in this study are aligned with those recommended in previous EDQM standardization studies to reduce assay variability [36]. The EDQM reference standard calibrated in IU was used in every assay plate to increase inter-assay comparability and to ensure reproducibility among laboratories, although it should be noted that the assays in this study were started prior to the calibration of the EDQM reference standard for the CHO cell assay [20]. Thus, the calibration unitage was based on the Histamine Sensitization Test study [30].

The present work demonstrated that the new xCELLigence system is a suitable tool to replace the subjective microscopy readout by measuring the change of cell-electrode impedance of CHO cells exposed to PT. The xCELLigence detected the PT-induced CHO cell clustering as a significant decrease in the cell index readouts compared to untreated CHO cells. Neutralization of PT activity with anti-PT neutralizing antibodies and testing of samples spiked with native PT demonstrated that the reduction in cell index readout is specifically related to the presence of PT activity. The assay threshold for a positive response by xCELLigence readout was determined by statistical comparison among cell control cell index and PT treatment cell index using a large set of experiments. To minimize the impact of day to day fluctuation for the initial cell numbers and growth rates (and hence cell index), sample and reference standard cell index were normalized against the cell controls (cell index values) within each experiment as a percentage response (cell control cell index represents 100% response). A positive PT response was defined as ≤ 65% of the cell control response based on the PT concentrations inducing significant or non-significant decrease of the cell index. Alignment between standard PT concentrations obtained by microscopy readout and xCELLigence analysis confirmed the suitability of the established xCELLigence threshold for a positive and negative response, and similar sensitivity of PT detection by both methods. The results for the gdPT samples unspiked or spiked with native PT tested across experiments performed on different days supported the use of the xCELLigence as a sensitive, robust and reproducible assay system to replace the microscopy readout of the CHO cell assay.

The xCELLigence system has been previously used in multiple applications such as cell proliferation, cell adhesion and morphology, compound-mediated cytotoxicity, cell-mediated cytotoxicity, and detection of viral cytopathic effect [39–57]. Although the xCELLigence system has been previously used to monitor cell interaction with other bacterial toxins such as Vibrio cholerae toxin [58] and Clostridium difficile toxin [59–62], to our knowledge, this is the first demonstration of the system suitability to detect PT. The sensitive CHO cell assay to detect residual pertussis toxin combined with the xCELLigence technology, that eliminates the subjectivity of the conventional microscope scoring, constitutes a novel methodology for testing and releasing acellular pertussis active ingredients.

The xCELLigence technology offers several advantages over conventional microscopy-based scoring of CHO cell clustering [7]. The system is now available in a multiple-plate formats (up to six 96-well E-plates can be run in parallel), which increases the assay throughput capacity. Automated cell index readouts save time and labor, provide objective archivable data and remove the subjectivity of the microscopy readout [19,20]. There is also the opportunity to reduce the xCELLigence assay reading time to earlier timepoints than 48 h, which could be explored once enough data have been collected at 48h to set acceptance criteria for the assay. The xCELLigence system software could be customized to provide specific data parameters (Relative cell index) that may assist with test validation. On the other hand, the xCELLigence system has the limitation that it cannot differentiate between PT-induced clustering and sample matrix induced cytotoxicity [7]. Thus, testing sample matrices in parallel by conventional microscopy readout might still be required during xCELLigence assay validation to confirm the absence of cytotoxicity observed at high sample concentration. Even so, the alignment of the assay parameters and results between CHO cell assay by microscopy and xCELLigence make the methods comparable to perform matrix verification studies in parallel when required.

In conclusion, the CHO cell assay with xCELLigence readout described herein offers an alternative automated method for evaluating PT-induced clustering in CHO cells and yields comparable results to the conventional microscopy assay. Furthermore, the assay can also be potentially used to test the neutralizing activity of anti-PT serum samples or monoclonal antibodies. The xCELLigence is well suited for an industrial application as it provides higher throughput readouts while eliminating the subjective microscopy evaluation. Robustness and validation of the xCELLigence method using a comprehensive set of assay parameters is required before the technology can be implemented for routine testing in quality control laboratories.

## Abbreviations

BRP PT: Biological reference preparation of pertussis toxin (standard PT)
C.C.: Control cells
CHO: Chinese Hamster Ovary
EDQM: European Directorate for the Quality of Medicine and Healthcare
gdPT: Genetically detoxified PT
IU: international units
PT: pertussis toxin
